# Dietary Risk Assessment of Cadmium Exposure Through Commonly Consumed Foodstuffs in Mexico

**DOI:** 10.3390/foods13223649

**Published:** 2024-11-16

**Authors:** Alejandra Cantoral, Sonia Collado-López, Larissa Betanzos-Robledo, Héctor Lamadrid-Figueroa, Betzabeth A. García-Martínez, Camilo Ríos, Araceli Díaz-Ruiz, Rosa María Mariscal-Moreno, Martha María Téllez-Rojo

**Affiliations:** 1 Health Department, Iberoamericana University, Mexico City 01219, Mexico; alejandra.cantoral@ibero.mx (A.C.); rosa.mariscal@ibero.mx (R.M.M.-M.); 2 Center for Nutrition and Health Research, National Institute of Public Health, Cuernavaca 62100, Mexico; mmtellez@insp.mx; 3 Doctoral Program in Epidemiology, Department of Public Health, National Autonomous University of Mexico, Mexico City 04510, Mexico; 4 Department of Perinatal Health, Center for Population Health Research, National Institute of Public Health, Cuernavaca 62100, Mexico; hlamadrid@insp.mx; 5 Service of Basic Neuroscience, National Institute of Rehabilitation, Mexico City 14389, Mexico; bgarcia@correo.xoc.uam.mx; 6 Research Direction, National Institute of Rehabilitation, Mexico City 14389, Mexico; camrios@yahoo.com.mx; 7 Department of Neurochemistry, National Institute of Neurology and Neurosurgery Manuel Velasco Suárez, Mexico City 14269, Mexico; adiaz@innn.edu.mx

**Keywords:** cadmium, health risk assessment, tolerable weekly intake, monitoring study, food contamination

## Abstract

Cadmium (Cd) is a toxic heavy metal widely distributed in foodstuffs. In Mexico, few studies have evaluated Cd content in foods. This study aimed to determine Cd concentrations in foodstuffs that are highly consumed and bought in Mexico City to identify foods exceeding the Maximum Level (ML) and to assess the health risks of theoretical Cd intake from a diet following the Mexican Dietary Guidelines. A total of 143 foodstuffs were analyzed by atomic absorption spectrophotometry. Theoretical Cd intake was estimated in portions per week and compared with the Cd Tolerable Weekly Intake (TWI = 2.5 μg/kg per body weight). A total of 68.5% of the foodstuffs had detectable Cd concentrations. Higher concentrations were found in oyster mushrooms (0.575 mg/kg), romaine lettuce (0.335 mg/kg), and cocoa powder (0.289 mg/kg). Food groups with higher mean concentrations were vegetables (0.084 mg/kg) and snacks, sweets, and desserts (0.049 mg/kg). Ancho chili and romaine lettuce exceed the ML. The theoretical Cd intake estimation was 1.80, 2.05, and 3.82 μg/kg per body weight for adults, adolescents, and school-age children, respectively. This theoretical Cd intake represents a health risk only for school children exceeding the TWI by 53.2%. Our study confirms the presence and risk of Cd in Mexican foodstuffs and highlights the importance of monitoring programs.

## 1. Introduction

Cadmium (Cd) is an environmental pollutant of increasing worldwide concern with numerous adverse health effects [1]. Cd occurs naturally in the environment in its organic form from sources such as volcanic emissions and rock erosion [2]. Additionally, the Cd level has increased as a consequence of human activities, such as production of batteries, electronic instruments, insecticides, fertilizers, and synthetic chemicals [3]. Consequently, it enters into the soil and water, and it can then be absorbed and accumulated in plants and animals, resulting in its consumption and absorption by the human body through the food chain [4,5]. 

The food supply is the primary source of Cd exposure for the non-smoking general population [6,7]. After dietary exposure, absorption of Cd is estimated to be low (3% to 5%); however, for children, it has been suggested that absorption can be as high as 44% [8]. Within the body, Cd is accumulated in different tissues and organs, particularly in the liver and kidneys [9]. Cd has a long biological half-life in the human body, estimated to be from 16 to 30 years, and chronic exposure to low concentrations of the metal is associated with kidney damage [10], some lung diseases, and high blood pressure [11] and is also a risk factor for osteoporosis [12]. Additionally, Cd has been classified as a human carcinogen by the International Agency for Research on Cancer [13].

Worldwide evidence documented that cereals, vegetables, nuts and pulses, starchy roots and potatoes, as well as meat and derivate products, contribute the most to human Cd exposure [14]. Other foods, such as chocolate and spices, also contain high concentrations of the metal [15,16]. The importance of the presence of Cd in food and the health risk repercussions has led agencies such as the Food and Agriculture Organization/World Health Organization (FAO/WHO) and the European Food Safety Authority (EFSA) Panel to establish the Maximum Levels (MLs) for foods [17] and the Tolerable Weekly Intake (TWI) of 2.5 μg/kg of Cd per body weight (BW) [18].

Additionally, another concern emerges when the foodstuffs recommended by Dietary Guidelines as a part of a healthy diet are also commonly reported with high Cd concentrations [19]. As an example, leafy vegetables, potatoes and grains, peanuts, soybeans, and sunflower seeds are foods with high nutritional properties; however, high concentrations of Cd have been reported in these products [7,18], which could negatively impact human health. In this sense, Cd food monitoring studies and the health risk assessments of its intake are important to ensure sufficient protection for consumers and human health by keeping the concentration of Cd intake as low as possible. However, in Mexico, there is no evidence of these types of studies.

Therefore, this study aimed to determine the Cd concentrations in commonly consumed foodstuffs by the Mexican population and purchased in Mexico City. Secondly, it aimed to identify foods and food groups that exceed the ML for Cd established by the FAO/WHO. Finally, it assessed the health risks of theoretical Cd intake according to a diet that follows the most recent Mexican Dietary Guidelines and compared the results with the Cd TWI.

## 2. Materials and Methods

A list of 100 foodstuffs most consumed by the Mexican population was identified for Cd concentration analysis using data from the National Health and Nutrition Survey 2018 (ENSANUT) and complemented with additional food items identified in the literature as sources of Cd [20]; therefore, a final sample of 143 was analyzed. Sample collection was performed in retail outlets from Mexico City from 12 April 2022 to 30 January 2023. The selected markets represent the most popular food purchase places (more details on the sample collection have already been published) [21].

Foodstuffs were analyzed at the Neurochemistry Laboratory of the National Institute of Neurology and Neurosurgery in Mexico City; more information on sample management before analysis has already been published [21]. The Cd concentrations were determined using an Atomic Absorption Spectrophotometer (AAS) (Perkin Elmer AAnalyst-600) equipped with a graphite furnace HGA-600 and coupled to an AS800 autosampler. The temperature programming for the method is shown in Table S1. The calibration curve solutions (0.5–2 μg/L) were prepared each day of the analysis by diluting a standard solution with 0.2% ultrapure HNO_3_ (Merck, Darmstadt, Germany), and the coefficient of determination was at least 0.99. Extrapolation was used to calculate the cadmium concentration in the sample. A volume of 20 μg/L of the acid digestion was injected directly into the graphite furnace. Cd concentrations were determined in duplicate for each sample. System validation tests were carried out following the recommendations established by the Commission for Analytical Control and Expansion of Coverage (CCAYAC-CR-03/0), and a repeatability test was performed. This test involves evaluating the lower limit of quantification (LoQ) by five-fold (0.5 μg/L, the lower value of the calibration curve) and three concentration values located within the calibration curve: low level 1.5 μg/L, medium level 3 μg/L, and high level 5 μg/L. For compliance with this test, the values obtained should have a maximum coefficient of variation of 20% for the LoQ and 15% for the other determinations. The results obtained were 13.9%, 3.6%, 7.8%, and 1.8%, respectively. Therefore, the proposed method complied with the repeatability test. We considered compliance through validation of the method used to perform duplicate testing. The standard addition technique was used to measure Cd levels for non-perishable foods, while direct acid digestion quantification (no standard added) was performed for perishable foods. The products with high concentrations were re-assessed, and if the values were above the maximum concentration of the calibration curve, the samples were diluted and re-assayed. As an internal control, on every day of analysis, a solution of acid digestion of bovine liver standard (similar digestion as the samples) equivalent to 5 μg/L of Cd was analyzed every 30 samples. A percentage recovery of 100.9 ± 13.6% was obtained from the controls analyzed.

### 2.1. Chemicals and Reagents Standard 

Solutions for Cd AAS (1000 μg/mL) were used as certified calibration standards (Perkin Elmer, Norwalk, CT, USA). Bovine Liver Standard NIST 1577c (Sigma-Aldrich, St. Louis, MO, USA) was used as the internal control. Nitric acid (HNO3) 65% Suprapur^®^ (Merck, Darmstadt, Germany) was used to prepare acid digestions and calibration curves. Dibasic ammonium phosphate (Sigma-Aldrich, St. Louis, MO, USA) and Triton X-100 (Sigma-Aldrich, St. Louis, MO, USA) were used to prepare the matrix modifier. All solutions were prepared with deionized water obtained from a Direct-Q 3 UV purification system (Millipore, Bedford, MA, USA).

### 2.2. Sample Treatment 

All samples were unpacked and inspected, and those that could decompose were stored at −20 °C until further experimentation to avoid decomposition. Foods packaged in paper or cardboard, plastic, or other containers were only cleaned with deionized water twice. The fruits were washed with soap and deionized water (twice). Those with inedible peel (such as bananas, mangoes, and oranges) were peeled, and only the pulp was used for the next steps. In the case of fruits such as apples (commonly consumed with the peel), the whole fruit was used. Meats (chicken, beef, pork) and eggs (without shell) were not cooked and were sampled raw for the next steps. The cereals and legumes were analyzed without processing (raw samples). The solid items underwent dehydration at 80 °C for 72 h, and the liquid samples were processed on a wet-weight basis. All solid items were ground using a house grinder and finally stored in polypropylene tubes until analysis. The digestion was carried out considering the recommendations outlined in Mexican Official NOM-117-SSA1-1994. The liquid and solid samples and the reference material were weighed in duplicate (0.1–0.2 g for solids) in test tubes, and 2 mL of 65% Suprapur^®^ HNO_3_ was added. The tubes were covered, mixed, and left at room temperature for 12 h. Subsequently, they were placed in a water bath (Labline instrument: shaking water bath) at 60 °C until a clear solution was obtained. Samples of 100 μL of acid digestion were placed directly into the autosampler for analysis. Those samples that presented concentrations higher than the calibration curve were diluted with 0.2% ultrapure HNO_3_.

### 2.3. Statistical Analysis

First, to perform the data characterization and summary, we averaged the Cd concentrations (mg/kg) obtained by duplicate per foodstuff analyzed and expressed as means and standard deviations (SD). All food items were encompassed in 14 food groups according to the Mexican Dietary Guidelines or to their nutritional characteristics [22,23]: (1) baby foods; (2) beverages; (3) cereals; (4) condiments and spices; (5) dairy; (6) fats and oils; (7) fruits; (8) legumes; (9) meats, sausages, and eggs; (10) nuts and seeds; (11) seafood; (12) snacks, sweets, and desserts; (13) soups and creams; and (14) vegetables. For each food group, we calculated the percentage of foodstuffs with non-detected Cd concentrations (<LoQ) and the percentage of foodstuffs with detected Cd concentrations (>LoQ). The mean, SD, and range (minimum and maximum) of Cd concentrations were estimated for each of the 14 food groups, considering only detected Cd values >LoQ.

Second, in order to identify the foodstuffs that exceeded the ML, the obtained Cd concentrations (mg/kg) were compared with the Cd ML in foods established by the Codex Alimentarius Commission of the FAO/WHO [17].

Finally, to perform the health risk assessment of Cd intake from Mexican foodstuffs, we determined the theoretical Cd intake in a diet that complies with the Mexican Healthy and Sustainable Guidelines recommendations [23]. Mexican guidelines established portion recommendations of intake according to age and sex population groups for the different food groups. On average, these guidelines recommend a daily intake of 4 to 5 portions of vegetables, 2 to 3 portions of fruits, 1 to 2 portions of legumes, 5 to 12 portions of cereals, and 1 to 2 portions of dairy, as along with weekly recommendations for the intake of food of animal origin, as part of a healthy diet. We obtained the average recommended number of portions per week for adults, adolescents, and school children. As a first step, we assigned at least one portion of each foodstuff analyzed per food group (considering a varied diet); as a second step, we considered those foods that are most consumed (i.e., maize) as a portion repeated throughout the week [24]. Then, we estimated the theoretical Cd intake (μg/per portion) for each portion of each food item and food group, considering moisture and fat changes that could occur during cooking [25]. 

Finally, for the health risk assessment analysis, we calculated the theoretical Cd intake for each age group, considering the average BW for adults, adolescents, and school children of 70 kg, 56 kg, and 25 kg, respectively [26]. Finally, we compared these results to the Cd TWI established by the EFSA of 2.5 μg/kg BW to ensure sufficient protection against Cd intake [18]. 

As an additional analysis, to ensure the robustness of our analysis, we employed Chauvenet’s criterion [27] to identify extreme values. To accomplish this, we compared the Cd concentrations obtained in our study with those reported from the U.S. [28] and the U.K. [29] (Supplementary Figure S1), and we excluded foodstuffs identified with extreme Cd concentrations from the theoretical Cd calculation.

All the analyses were conducted using STATA 17 statistical software (StataCorp LLC, College Station, TX, USA).

## 3. Results

### 3.1. Cadmium Concentrations in Mexican Foodstuffs 

A total of 143 foodstuffs were analyzed, and the detected Cd (>LoQ) concentrations were identified in 68.5% (*n* = 98), ranging from 0.004 mg/kg to 0.575 mg/kg. We identified 45 foodstuffs below the LoQ. The top ten foodstuffs with the highest Cd concentrations were oyster mushrooms “*Pleorotus ostreatus*” (0.575 mg/kg), romaine lettuce “*Lactuca sativa* L. var*. longifolia*“ (0.335 mg/kg), cocoa powder (0.289 mg/kg), chocolate powder (0.117 mg/kg), saladette tomatoes “*Solanum lycopersicum roma*” (0.095 mg/kg), breadcrumbs (0.069 mg/kg), chocolate bars (0.060 mg/kg), ancho chilies “*Capsicum annuum*” (0.059 mg/kg), chard “*Beta vulgaris* var*. cicla*” (0.058 mg/kg), and mushrooms “*Agaricus bisporus*” (0.055 mg/kg). All these items came from vegetable sources. The complete list of the 143 foodstuff samples evaluated is presented in Table S2.

Table 1 shows the average estimate of Cd concentration by food group. Almost all food groups contain foodstuffs with detected Cd concentrations, from 22 to 100% of the analyzed samples per group. The food groups with the highest percentage of detected Cd concentrations were seafood, the food group with 100% detected Cd concentrations, followed by cereals (96%, n = 23), vegetables (89%, n = 17), legumes (87%, n = 7), condiments and spices (82%, n = 9), snacks, sweets, and deserts (78%, n = 14), and baby foods (75%, n = 9). 

From these food groups with detectable Cd concentrations, higher means were found in vegetables [0.084 mg/kg (range: 0.006–0.575 mg/kg)], followed by snacks, sweets, and desserts [0.049 mg/kg (range: 0.004–0.289 mg/kg)], fruits [0.037 mg/kg (range: 0.016–0.050 mg/kg)], condiments and spices (0.028 mg/kg [range: 0.009–0.059 mg/kg]), and cereals [0.026 mg/kg (range: 0.005–0.069 mg/kg)]. The lowest detected mean Cd concentrations were found in the food groups of fats and oils [0.008 mg/kg (range: 0.008–0.009 mg/kg)], legumes [0.009 mg/kg (range: 0.006–0.017)], and baby foods [0.010 mg/kg (range: 0.004–0.023)]. The only food group with no detected concentrations was dairy, for which all items were below the LoQ.

### 3.2. Mexican Foodstuffs That Exceed the Food and Agriculture Organization/World Health Organization Maximum Level (ML) Established for Cadmium

In Table 2, we observed that, of the 98 foodstuffs with detectable Cd concentrations, 61 had a reference value of ML established by the FAO/OMS. Of these 61 foodstuffs, two exceeded the ML: ancho Chili “*Capsicum annuum*” (0.059 mg/kg) and romaine lettuce “*Lactuca sativa* L. var*. longifolia*” (0.335 mg/kg) Broccoli “*Brassica oleracea* var. *italica*” (0.046 mg/kg) was within the limited concentration. In addition, it is important to highlight that foodstuffs such as saladette tomatoes “*Solanum lycopersicum*”, mushrooms “*Agaricus bisporus*” and “*Pleorotus ostreatus*” have no reference ML values to compare, and both foodstuffs presented the highest Cd concentrations in this study. 

### 3.3. Health Risk Assessment of Theoretical Cadmium Intake Based on the Adherence to the Healthy and Sustainable Dietary Guidelines for the Mexican Population 

**Figure 1:**
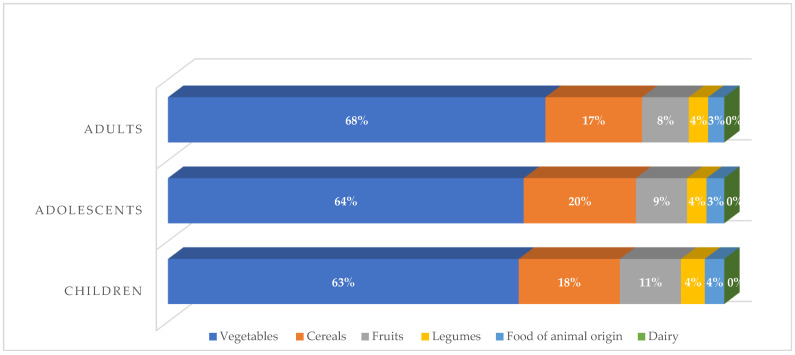
Contribution per food group to the total theoretical dietary weekly cadmium intake by age group, following the recommendations of the Healthy and Sustainable Guidelines for the Mexican population.

Table 3 and Figure 1 present the results of the estimated theoretical Cd intake per kg of BW for adults, adolescents, and school children, following the recommended weekly food portions per food group in the Mexican Healthy and Sustainable Dietary Guidelines.

Following this guideline would result in a weekly theoretical Cd intake of 1.80 μg/kg of BW, 2.06 μg/kg per BW, and 3.83 μg/kg per BW for adults, adolescents, and school children, respectively. Compared to the TWI (2.5 μg/kg per BW), this theoretical Cd intake does not represent a health risk for adults and adolescents, but for school children, a theoretical Cd intake of 3.83 μg/kg of BW exceeds the TWI by 53.2% (Table 3).

The food groups with the highest contribution to the theoretical Cd intake in all age groups were vegetables and cereals, with more than 80% of the combined contribution (Figure 1).

It is important to mention that these guidelines do not include unhealthy foods with detectable Cd concentrations, such as chocolate powder (0.117 mg/kg), chocolate bars (0.060 mg/kg), dark chocolate bars (0.029 mg/kg), potato chips (0.035 mg/kg), pastries (0.028 mg/kg), and different candies (average 0.011 mg/kg). Therefore, including these foodstuffs in the theoretical Cd intake would imply a greater risk of exceeding Cd TWI, with prolonged consumption potentially adversely affecting health.

### 3.4. Additional Analysis: Comparison Between Cd Concentrations Detected in Mexican Foodstuffs with Total Diet Studies from the U.S. and the U.K.

Among the 143 Mexican foodstuffs that were analyzed, we found 79 that were comparable to those reported by TDSs in the U.S. or U.K. (between 77 and 54 were equivalent to the U.S. and U.K. data, respectively). Overall, Mexico had a high Cd concentration in many foodstuffs (Figure S1). In the U.S., the highest Cd concentrations were identified in sunflower seeds (0.333 mg/kg), spinach (0.222 mg/kg), lettuce (0.061 mg/kg), and French fries (0.058 mg/kg). In Mexico, sunflower seeds had Cd concentrations <LoQ, while spinach (0.050 mg/kg) and French fries (0.042 mg/kg) showed lower concentrations compared to the U.S., but lettuce presented a higher concentration (0.335 mg/kg). For the U.K., the highest Cd concentrations were reported in potato chips (0.078 mg/kg), chicken liver (0.032 mg/kg), French fries (0.031 mg/kg), and flour (0.030 mg/kg). In Mexico, lower concentrations were identified in potato chips (0.035 mg/kg) and chicken liver (0.012 mg/kg), but higher concentrations were found in French fries (0.042 mg/kg) and wheat flour (0.036 mg/kg). Consistently, romaine lettuce, French fries, potato chips, potatoes, wheat flour, whole wheat bread, breakfast cereals, white bread, canned tuna, dark chocolate bars, sweet cookies, carrots, oatmeal, and pastries were identified as relevant sources of Cd concentrations across all three countries. 

## 4. Discussion

This study is the first effort to evaluate Cd concentrations in commonly consumed Mexican foodstuffs and the possible health risks associated with their consumption. We found that 68.5% (n = 98) of the foodstuff samples had detectable concentrations of Cd, with concentrations ranging between 0.004 mg/kg and 0.575 mg/kg. As expected, crop-sourced food items such as mushrooms, lettuce, tomato, and cocoa presented the highest Cd concentrations. Concerning mushrooms, these results are not surprising. It is documented that they have a very effective mechanism to accumulate metals such as Cd from the environment [30]. In the case of lettuce, Cd possesses a high capacity of being transferred from the soil to the leaves [31]. With respect to cocoa, a product derived from cacao, consistent literature reports that cacao beans tend to bioaccumulate Cd [32], which is related to environmental factors and the chemical composition of cacao, which allows strong binding of Cd in cacao tissues and cocoa products. This has become a global concern; a review on ML of Cd in cocoa reported high Cd levels in the main Latin American producing countries. In addition, a study that assessed the concentration of different elements, including Cd, in 155 chocolate samples from the U.S. market identified high mean concentrations of Cd in samples from Central America countries (which included five samples from Mexico), where cocoa Cd concentrations were found to be as high as 689 µg/kg [33]. 

In terms of food groups, we identified that vegetables (0.084 mg/kg), snacks, sweets and desserts (0.049 mg/kg), and cereals (0.025 mg/kg) were the food groups with the highest Cd concentrations; additionally, cereals were the food group with the most items with detectable Cd concentrations (96%). Consistently cereal crops such as rice, wheat, and maize were identified as major sources of Cd exposure for humans. Leading to extensive efforts to understand the mechanisms of Cd accumulation in these foods, specific genes and transporters involved in Cd uptake and transport have been identified in rice, with similar pathways observed in wheat [34]. 

Similar to our results, diverse studies have shown that Cd concentrations in vegetable and cereal groups are the main contributors to Cd intake. Using data from the National Health and Nutrition Examination Survey (NHANES), a group of researchers in the United States found that the food groups that contributed the most to Cd intake were cereals and bread (34%), leafy vegetables (20%), potatoes (11%), legumes and nuts (7%), and stem/root vegetables (6%) [7]. EFSA`s reports state that cereals and cereal products, vegetables, nuts and pulses, starchy roots and potatoes, as well as meat and meat products, are the food groups that contribute the most to human Cd exposure [18]. In China, cereals (46.2%), vegetables (19.2%), and aquatic food (18.4%) contributed the most to the dietary Cd exposure of the Chinese population [35]. In addition, in a study of the Shenzhen population, vegetables were identified as the principal food group contributor to dietary Cd exposure at 32.6%, followed by rice and its products (19.2%), fish, seafood, and shellfish (18.5%), and legumes, nuts, and their products (14.5%) [36]. Similarly, in a contaminated area of Thailand, the major food groups identified as the principal contributors to Cd exposure were rice and grains, shellfish and seafood, meat, and vegetables [37]. 

In the present analysis the dairy group was the only food group with no detected Cd concentrations. Similarly, a report from Egypt estimated that raw milk contributed only 1.59% of the estimated weekly intake [38]. Moreover, in Peru, 19 milk samples were evaluated, and the Cd concentrations were low (0.007 ± 0.006 mg/kg), with the intake at all ages being below the estimated Cd TWI [39]. A review conducted in China identified 13 studies that evaluated Cd concentrations in milk and milk products, showing contrasting results, highlighting that Cd concentrations in milk in China were lower than those reported in milk from other developing countries but were higher than those in developed countries [40].

We identified one previous analysis in Mexico concerning dietary Cd sources. The authors identified potential dietary contributors of Cd, such as chorizo (mean 0.0302 mg/kg), sausages (mean 0.0032 mg/k), ham 0.0056 mg/kg), and chicken breast (0.0007 mg/kg). Similarly, in this analysis, we also identified Cd in chorizo (sausage “longaniza”) (mean 0.012 mg/kg), in turkey sausages (Brand 2) (0.015 mg/kg), and in two brands of pork ham (mean 0.011 mg/kg), but not in chicken breast (<LoQ) [41]. 

The vegetable food group was the food group with the highest Cd values, as many vegetables had very high Cd concentrations. This highlights the need to maintain a varied diet within the food groups to reduce the daily intake of the foods identified with the highest concentration. A previous cohort study in Mexico City identified that refined grains and vegetables contributed the most to dietary Cd intake for pregnant women and children, with over 60% of the vegetable contribution coming from leafy greens [42]. This result is similar to our estimation, where over 60% of the Cd contribution came from the vegetable group.

Concerning the Cd intake results, a diet that follows the adherence to the Mexican Healthy and Sustainable Guidelines would result in a weekly theoretical Cd intake below the TWI for both adults and adolescents (1.80 μg/kg BW and 2.05 μg/kg BW, respectively), and above the TWI for school-age children (3.82 μg/kg of BW). According to another study, with information from individuals aged 2 years and older from the National Health and Nutrition Examination Survey (NHANES) 2007–2012, the average dietary Cd consumption in the U.S. general population was 0.54 μg/kg BW/week (22% of the TWI) [7], which was lower than the levels found in our study [7]. Additionally, data from the sixth Chinese TDS reported a mean Cd intake for male adults of 8.26 μg/kg BW per month (2.60–30.02 μg/kg BW per month), which would represent a Cd intake of 2.07 μg/kg BW per week, which is higher than our estimated Cd intake for adults (1.80 μg/kg BW/week) [43].

Regarding the intake results for children, another study in the U.S. found that the Cd intake in infants and young children who regularly consumed rice, spinach, oats, barley, potatoes, and wheat did not exceed the daily intake set by the EFSA but exceeded the Cd exposure set by the Agency for Toxic Substances and Disease Registry (ATSDR), which is considered a stricter level [44]. 

Similar to our results, a cohort study based in Mexico City evaluated children’s dietary Cd intake using data from various countries on Cd concentrations in foods. The study reported Cd intakes of 4.43 ± 2.53 μg/d, 4.65 ± 2.45 μg/d; 6.00 ± 3.45 μg/d; 6.83 ± 3.15 μg/d and 8.09 ± 4.33 μg/d at 1, 2, 4, 6 and 9 years of age, respectively. It also identified leafy greens as top contributors at ages 1 and 2 (1 year: 16.0%, 2 years: 9.0%); at age 4, sweets (6.8%); at age 6, lettuce (6.8%) and sweets (5.5%). Lastly, at age 9, the major contributors were lettuce (6.0%), pasta soup (5.7%), and sweets (5.5%) [45]. Moreover, a previous study in Mexico indicated that 16–64% of children exceed the TWI at ages 1 to 9 years [45]. 

In our study, vegetables, and cereals were the food groups with the highest contribution to the theoretical Cd intake in a diet adhering to the Mexican Healthy and Sustainable Diet Guidelines across all age groups, with more than 80% of the combined contribution. This is consistent with the major sources of Cd reported in the U.S., where cereals and bread represent 34% and leafy vegetables 20% [7]. Vegetable consumption is recommended because they are a source of vitamins, fiber, and bioactive components; however, the increasing evidence of the accumulation of toxic metals such as Cd requires routine monitoring and highlights the recommendation of consuming a varied diet to avoid excess exposure to a particular foodstuff [46].

Our study has important limitations. First, we did not have a representative foodstuff sample, as all samples were bought from a specific area of Mexico City. In addition, a random selection of stores and foodstuffs was not carried out, so the variability in Cd content in foods from different regions of the country, as well as variations due to seasonality, could not be ascertained. Second, we had a small sample size of the analyzed foodstuffs. Third, our Cd concentrations were measured by AAS, and these results differ from other methods, such as ICP-MS. Third, the dietary theoretical Cd estimation was based on a diet that follows the Healthy and Sustainable Mexican Guidelines recommendations and differed from those obtained using other methods reported in the literature that based Cd intake estimations on Food Frequency Questionaries. It is important to mention that these guidelines do not include unhealthy foods; therefore, our theoretical Cd intake does not include foodstuffs “not recommended or unhealthy”, such as the snacks, sweets, and desserts group, which was the second food group with the highest mean Cd concentrations (0.049 mg/kg). In our results, this group included items with detectable Cd concentrations, such as chocolate powder (0.117 mg/kg), chocolate bars (0.060 mg/kg), dark chocolate bars (0.029 mg/kg), potato chips (0.035 mg/kg), pastries (0.028 mg/kg), and different candies (average 0.011 mg/kg). Therefore, including these foodstuffs in the estimation of theoretical Cd intake would imply a greater risk of exceeding Cd TWI, whose prolonged consumption could adversely affect health, especially for young children, because of their higher absorption and lower detoxification capacities [47]. 

## 5. Conclusions

Our study confirms that foodstuffs bought from retail stores in Mexico City are an important source of Cd intake and could represent a health risk for school children. This highlights the importance of monitoring the presence of contaminants in the foods consumed by the population, especially in those widely recommended by the Healthy and Sustainable Dietary Guidelines. The frequent consumption of those foods with detectable Cd concentrations could be considered chronic exposure, which is particularly important for children. Therefore, food quality and safety are among the most important public health concerns, and the food available on the market should be free of chemical contaminants that pose a risk to consumer health. Food safety is not only the responsibility of food producers but also of state governments and agencies, which should systematically monitor their safety as an essential strategy for public health actions. 

## Figures and Tables

**Table 1:** Cadmium concentrations (mg/kg) by food group analyzed (n = 143).

Food Group	Foodstuffs Analyzed	Number of Foodstuffs Analyzed (n)	Foodstuffs with Non-Detectable Cd Concentrations n (%)	Foodstuffs with Detectable Cd Concentrations n (%)	Mean (SD) of Detectable Cd Concentrations (mg/kg)	Range of Detectable Cd Concentrations (min–max)
Baby foods	Apple Juice; Carrot Porridge, Chicken, Vegetables and Rice Porridge; Infant Formula Soy Milk (Brand 1); Infant Formula Soy Milk (Brand 2); Infant Formula Whole Milk (Brand 1); Infant Formula Whole Milk (Brand 2); Infant Formula Whole Milk (Brand 3); Infant Grow and Gain Strawberry Shake; Infant Rice Cereal (Brand 1); Infant Rice Cereal (Brand 2); Strawberry and Apple Cereal Snack.	12	3 (25%)	9 (75%)	0.010 (0.002)	0.004–0.023
Beverages	Bottled Soft Drink; Soluble Coffee.	2	1 (50%)	1 (50%)	na	na
Cereals	Amaranth “*Amaranthus* spp.”; Breadcrumbs; Breakfast Cereal; Maize “*Zea mays*”; Maize grain “*Zea mays*”; Maize Flour; Crackers; French Fries; Oat “*Avena sativa*”; Potato “*Solanum tuberosum*”; Pre-Cooked Rice; Rice “*Oryza sativa*” (Brand 1); Rice “*Oryza sativa*”(Brand 2); Rice “*Oryza sativa*” (Brand 3); Rice Cake; Rice Flour; Wheat Cookies; Wheat Flour (Brand 1); Wheat Flour (Brand 2); Wheat Tortillas; White Bread (Bakery); White Bread (Brand 1); Whole Wheat Bread (Bakery); Whole Wheat Bread (Brand 1).	24	1 (4%)	23 (96%)	0.026 (0.002)	0.005–0.069
Condiments and spices	Ancho Chilies “*Capsicum annuum*”; Black Pepper “*Piper nigrum L*”; Canned Green Chilies; Chicken Broth Cubes; Chili Powder; Chilies “Capsicum annuum ‘Guajillo”; Guajillo Industrialized Sauce; Mole; Paprika “*Capsicum annuum*”; Saffron “*Crocus sativus*”; Turmeric “*Curcuma longa*”.	11	2 (18%)	9 (82%)	0.028 (0.004)	0.009–0.059
Dairy	Asadero Cheese; Manchego Cheese; Natural Yogurt; Petit Suisse; Whole Liquid Milk (Brand 1); Whole Liquid Milk (Brand 2).	6	6 (100%)	0 (0%)	na	na
Fats and oils	Hass Avocado “*Persea americana*”; Butter; Lard; Margarine; Mayonnaise; Sour Cream (Brand 1); Sour Cream (Brand 2); Vegetable Oil (Brand 1); Vegetable Oil (Brand 2).	9	7 (78%)	2 (22%)	0.008 (0.001)	0.008–0.009
Fruits	Golden Yellow Apple “*Malus domestica ‘golden delicious’*”; Grape “*Vitis vinifera*”; Grapefruit “*Citrus paradisi*”; Guava “*Psidium guajava*”; Lime “*Citrus autantifolia*”; Melon “*Cucumis melo*”; Orange “*Citrus sinensis L.*”; Strawberry “*Fragaria x anassa*”; Ataulfo Mango “*Mangifera indica ‘Ataulfo’*”; Manila Mango “*Mangifera indica ‘Manila*”; Banana Tabasco “*Musa × paradisiaca*”; Papaya “*Carica papaya*”; Watermelon “*Citrullus lanatus*”.	13	10 (77%)	3 (23%)	0.037 (0.006)	0.016–0.050
Legumes	Black Beans “*Phaseolus vulgaris*”; Black Canned Beans; Chickpeas “*Cicer arietinum*”; Fava Beans “*Vicia faba*”; Lentils “*Lens culinaris*”; Lentils Instant Soup; Soybean “*Glycine max* L*. Merr.*”; White Beans “*Phaseolus vulgaris*”.	8	1 (13%)	7 (87%)	0.009 (0.002)	0.006–0.017
Meats, sausages and eggs	Beef (Brand 1); Beef (Brand 2); Chicken, Chicken Liver, Eggs (Brand 1); Eggs (Brand 2); Pork (Brand 1); Pork (Brand 2); Pork Ham (Brand 1); Pork Ham (Brand 2); Sausage “Longaniza”; Turkey Sausages (Brand 1); Turkey Sausages (Brand 2).	13	6 (46%)	7 (54%)	0.011 (0.002)	0.006–0.015
Nuts and seeds	Peanuts; Sunflower Seeds.	2	1 (50%)	1 (50%)	na	na
Seafood	Canned Tuna (Brand 1); Canned Tuna (Brand 2); Fresh Tuna.	3	0 (0%)	3 (100%)	0.016 (0.001)	0.010–0.019
Snacks, sweets and desserts	Chamoy Candy; Chewing Gum; Chocolate Bar; Chocolate Powder; Cocoa Powder; Dark Chocolate Bars; Honey; Jelly; Marzipan Candy; Pastries; Popsicle; Potato Chips (Brand 1); Potato Chips (Brand 2); Sweet Cookie; Tamarind Candy; Tamarind Poblano Candy; Tamarind Popsicle; Wheat Chips.	18	4 (22%)	14 (78%)	0.049 (0.005)	0.004–0.289
Soups	Canned Vegetable Soup; Instant Pasta Soup; Pasta Soup to Prepare.	3	1 (33%)	2 (67%)	0.032 (0.003)	0.030–0.033
Vegetables	Broccoli “*Brassica oleracea* var*. italica*”; Cabbage “*Brassica oleracea* var*. capitata L.*”; Carrot “*Daucus carota subsp. sativus*”; Cauliflower “*Brassica oleracea* var*. botrytis*”; Chard “*Beta vulgaris* var*. cicla*”; Chayote “*Sechium edule*”; Coriander “*Coriandrum sativum*”; Cucumbers “*Cucumis sativus*”; Fresh Green Chilies “*Capsicum annuum ‘Serrano’*”; Green Beans “*Phaseolus vulgaris*”; Jicama “*Pachyrhizus erosus*”; Mushrooms “*Agaricus bisporus*”; Nopal Cactus “*Opuntia ficus-indica L.*”; Romaine Lettuce “*Lactuca sativa l.* var*. longifolia*”; Saladette Tomatoes “*Solanum lycopersicum*”; Oyster Mushrooms “*Pleorotus ostreatus*”; Spinach “*Spinacia oleracea*”; White Onions “*Allium cepa*”; Zucchini “*Cucurbita pepo* L.”.	19	2 (11%)	17 (89%)	0.084 (0.011)	0.006–0.575

Abbreviations: Cd: cadmium; na: not applicable because the value corresponds to a concentration <LoQ. Non-detectable concentrations were defined as concentrations below the limit of quantification (<LoQ). Detectable concentrations were defined as concentrations above the limit of quantification (>LoQ).

**Table 2:** Comparison between cadmium concentrations in Mexican foodstuffs and the Food and Agriculture Organization/World Health Organization Maximum Level established for cadmium (*n* = 61).

Mexican Foodstuffs Analyzed per Food Group	Cd Mean Concentration (mg/kg)	FAO/WHO Product Name Classification	FAO/WHO ML for Cd (mg/kg)
Baby foods			
Carrot Porridge	0.004	Root and tuber vegetables ^	0.1
Infant Rice Cereal (Brand 1)	0.016	Rice, Polished	0.4
Infant Rice Cereal (Brand 2)	0.023	Rice, polish	0.4
Strawberry and Apple Cereal Snack	0.013	Cereal grains	0.1
Cereals			
Amaranth “*Amaranthus* spp.”	0.019	Cereal grains	0.1
Breadcrumbs	0.069	Wheat ^	0.2
Breakfast Cereals	0.008	Cereal grains	0.1
Crackers	0.011	Wheat ^	0.2
French Fries (Street Stand)	0.042	Root and tuber vegetables ^	0.1
Maize Flour	0.005	Cereal grains	0.1
Maize “*Zea mays*”	0.007	Cereal grains	0.1
Oat “*Avena sativa*”	0.005	Cereal grains	0.1
Pre-Cooked Rice	0.017	Rice, polish	0.4
Potato “*Solanum tuberosum*”	0.009	Root and tuber vegetables	0.1
Rice Cake	0.024	Rice, polish ^	0.4
Rice Flour	0.037	Rice, polish ^	0.4
Rice “*Oryza sativa*” (Brand 1)	0.030	Rice, polish	0.4
Rice “*Oryza sativa*” (Brand 2)	0.027	Rice, polish	0.4
Rice “*Oryza sativa*” (Brand 3)	0.031	Rice, polish	0.4
Wheat Flour (Brand 1)	0.032	Wheat	0.2
Wheat Flour (Brand 2)	0.040	Wheat	0.2
Wheat Tortillas	0.028	Wheat	0.2
Wheat Cookies	0.033	Wheat ^	0.2
White Bread (Brand 1)	0.033	Wheat ^	0.2
White Bread (Bakery)	0.037	Wheat ^	0.2
Whole Wheat Bread (Bakery)	0.037	Wheat ^	0.2
Whole Wheat Bread (Brand 1)	0.033	Wheat ^	0.2
Condiments and spices			
Ancho Chili “*Capsicum annuum*”	**0.059**	Fruiting vegetable	**0.05**
Guajillo Chili “*Capsicum annuum ‘Guajillo’*”	0.018	Fruiting vegetable	0.05
Legumes			
Black Beans “*Phaseolus vulgaris*”	0.017	Pulses	0.1
Black Canned Beans	0.007	Pulses	0.1
Chick Beans “*Cicer arietinum*”	0.008	Pulses	0.1
Fava Bean “*Vicia faba*”	0.009	Pulses	0.1
Lentils “*Lens culinaris*”	0.006	Pulses	0.1
Lentils Instant Soup	0.007	Pulses	0.1
Soybean “*Glycine max* L*. merr.*”	0.009	Pulses	0.1
Snacks, sweets, and desserts			
Cocoa Powder	0.289	Chocolate ≥70% total cocoa solids	0.9
Chocolate Bars	0.060	Chocolate containing or declaring ≥ 50% to < 70% total cocoa solids	0.8
Chocolate Powder	0.117	Chocolate containing or declaring ≥ 50% to < 70% total cocoa solids	0.8
Dark Chocolate Bars	0.029	Chocolate containing or declaring ≥ 50% to < 70% total cocoa solids	0.8
Pastries	0.028	Wheat ^	0.2
Potato Chips (Brand 1)	0.029	Root and tuber vegetables ^	0.1
Potato Chips (Brand 2)	0.041	Root and tuber vegetables ^	0.1
Sweet Cookies	0.018	Wheat ^	0.2
Wheat Chips	0.011	Wheat ^	0.2
**Soups**			
Instant Pasta Soup	0.030	Wheat	0.2
Pasta Soup to Prepare	0.033	Wheat	0.2
**Vegetables**			
Broccoli “*Brassica oleracea* var*. italica*”	0.046	Brassica vegetables	0.05
Cabbage “*Brassica oleracea* var*. capitata* L.”	0.007	Brassica vegetables	0.05
Cauliflower “*Brassica oleracea* var. *botrytis*”	0.036	Brassica vegetables	0.05
Carrot “*Daucus carota subsp. sativus*”	0.016	Root and tuber vegetables	0.1
Chard “*Beta vulgaris* var*. cicla*”	0.058	Leafy vegetables	0.2
Coriander “*Coriandrum sativum*”	0.023	Leafy vegetables	0.2
Cucumber “*Cucumis sativus*”	0.041	Fruiting vegetable	0.05
Fresh Green Chilies “*Capsicum annuum ‘Serrano’*”	0.014	Fruiting vegetable	0.05
Green Beans “*Phaseolus vulgaris*”	0.006	Legume vegetables	0.1
Nopal Cactus “*Opuntia ficus-indica* L.”	0.016	Leafy vegetables ^	0.2
Romaine Lettuce “*Lactuca sativa* L. var*. longifolia*”	**0.335**	Leafy vegetables	**0.2**
Spinach “*Spinacia oleracea*”	0.059	Leafy vegetables	0.2
White Onions “*Allium cepa*”	0.022	Bulb vegetable	0.05
Zucchini “*Cucurbita pepo* L.”	0.025	Fruiting vegetables	0.05

Abbreviations: ML: Maximum Level; Cd: cadmium; FAO: Food and Drug Administration; WHO: World Health Organization. ^ Foods were grouped into this category based on their nature, even though these specific foods are not detailed by the FAO/WHO product name classification. Source: FAO and WHO General Standard for Contaminants and Toxins in Food and Feed. Note: FAO/WHO product name classification of fruiting vegetables does not apply to tomatoes and edible fungi. Cd concentrations in bold exceed ML.

**Table 3:** Calculation of the theoretical cadmium intake per age group according to average body weight, following the recommended weekly portions in the Healthy and Sustainable Guidelines for the Mexican Population.

	Theoretical Cd Intake for Adults			Theoretical Cd Intake for Adolescents			Theoretical Cd Intake for School Children		
Food Group	Recommended Portions per Week	Cd Intake (μg/per Portion)	Theoretical Cd Intake per 70 kg of BW	Recommended Portions per Week	Cd Intake (μg/per Portion)	Theoretical Cd Intake per 56 kg of BW	Recommended Portions per Week	Cd Intake (μg/per Portion)	Theoretical Cd Intake per 25 kg of BW
Vegetables	31.5	85.415	1.220	31.5	73.627	1.315	28.0	60.267	2.411
Fruits	14.0	10.560	0.151	21	10.560	0.189	21.0	10.560	0.422
Legumes	10.5	4.418	0.063	10.5	4.068	0.073	10.5	4.068	0.163
Cereals	59.5	21.996	0.314	66.5	23.317	0.416	49.0	17.427	0.697
Dairy	10.5	0.000	0.000	10.5	0.000	0.000	7.0	0.000	0.000
Food of animal origin									
Beef	3.5	0.525	0.008	3.5	0.525	0.009	3.0	0.450	0.018
Other red meat	3.0	0.000	0.000	3.0	0.000	0.000	2.5	0.000	0.000
Chicken	9.0	0.000	0.000	8.0	0.000	0.000	6.5	0.000	0.000
Seafood	3.5	2.073	0.030	3.5	2.073	0.037	3.5	2.073	0.083
Eggs	7.0	1.078	0.015	7.0	1.078	0.019	5.0	0.770	0.031
Total per week		**126.07 μg**	**1.80 μg/kg per BW**		**115.25 μg**	**2.06 μg/kg per BW**		**95.62 μg**	**3.83 μg/kg per BW**

## Data Availability

The data presented in this study are available on request from the corresponding author. The data are not publicly available due to privacy restrictions.

## Supplementary Materials

The following supporting information can be downloaded at: https://www.mdpi.com/article/10.3390/foods13223649/s1, Table S1: Instrumental condition used in the analysis of Cd, Table S2: Complete information for each of the Mexican Foodstuffs analyzed for the determination of Cd concentrations (n = 143); Figure S1: Comparison of cadmium concentrations (mg/kg) between Mexico and reported Cadmium concentrations in U.S and U.K total diet studies (n = 79).

## Abbreviations

BW	Body Weight
Cd	Cadmium
EFSA	European Food Safety Authority
EU	European Union
FDA	Food and Drug Administration
FAO	Food and Agriculture Organization of the United Nations
JECFA	Joint FAO/WHO Expert Committee on Food Additives
MLs	Maximum Levels of contaminants and toxins in food
NHANES	National Health and Nutrition Examination Survey
PTMI	Provisional Tolerable Monthly Intake
TDS	Total Diet Study
TWI	Tolerable Weekly Intake
WHO	World Health Organization
